# Resveratrol alleviates functional dyspepsia-like pathology by modulating chemokine- and cytokine-related immune–epithelial signaling in the duodenal mucosa

**DOI:** 10.3389/fimmu.2026.1906024

**Published:** 2026-08-13

**Authors:** Yang Lv, Wei Gong, Long Li, Zeli Li, Jiarui Nicole Fu, Fachao Zhi, Shanshan Cai

**Affiliations:** 1 Department of Gastroenterology, Shenzhen Hospital, Southern Medical University, Guangdong, China; 2 Department of Gastroenterology, Guangdong Provincial Key Laboratory of Gastroenterology, Institute of Gastroenterology of Guangdong Province, Nanfang Hospital, Southern Medical University, Guangdong, China; 3 Nutrition and Dietetic Services, Gloucestershire Royal Hospital, NHS Foundation Trust, National Health Service (NHS), Gloucester, United Kingdom; 4 Dongguan Hospital of Guangzhou University of Chinese Medicine, Dongguan Traditional Chinese Medicine Hospital, Guangdong, China; 5 Division of Biosciences, Faculty of Life Sciences, University College London, London, United Kingdom; 6 Department of Biological Chemistry, University of Michigan Ann Arbor, Michigan, MI, United States; 7 Immunology Research Laboratory, Department of Research and Development, PyraGeo Biosciences Co., Ltd., Changsha, China; 8 Pharmacology Research Laboratory, Department of Research and Development, PyraGeo Biosciences Co., Ltd., Changsha, China

**Keywords:** epithelial barrier, functional dyspepsia, gut microbiota, immune–epithelial signaling, ligand–receptor signaling, mast cells, resveratrol

## Abstract

**Objective:**

Resveratrol is a dietary polyphenol found in grapes, peanuts, and berries, with reported anti-inflammatory, immunomodulatory, barrier-protective, and microbiota-regulating properties. Functional dyspepsia (FD) is associated with duodenal immune dysregulation, epithelial barrier impairment, and microbial imbalance, suggesting an active mucosal microenvironment. This study therefore examined whether resveratrol could alleviate FD-like pathological changes. We also examined its links with epithelial homeostasis, gut microbiota, and chemokine- or cytokine-related ligand–receptor signaling.

**Methods:**

An FD-like mouse model was established and treated with resveratrol. Gastric emptying was assessed, and duodenal histopathology was examined by hematoxylin–eosin staining, including epithelial injury, goblet cell alterations, crypt disorganization, and inflammatory cell infiltration. Toluidine blue staining assessed mast cell accumulation. RNA sequencing, GO/KEGG enrichment, and protein–protein interaction analyses characterized immune-related pathways, hub genes, and potential signaling networks. qPCR validated genes related to mucosal metabolism and epithelial function, chemokine- and cytokine-related ligand–receptor signaling genes, mast cell markers, and epithelial barrier genes. 16S rRNA sequencing assessed gut microbial changes.

**Results:**

FD-like mice showed delayed gastric emptying, duodenal mucosal injury, inflammatory cell infiltration, goblet cell alterations, crypt disorganization, and mast cell accumulation, indicating low-grade mucosal immune activation and epithelial barrier disturbance. Resveratrol improved gastric emptying, alleviated duodenal injury, and reduced mast cell infiltration. Transcriptomic analysis showed broad changes in immune-related programs, particularly those associated with antigen presentation, adaptive immune responses, pro-inflammatory signaling, chemokine and cytokine activity, and mucosal immune regulation. PPI and qPCR analyses further supported the regulation of mucosal homeostasis, candidate *Ccl2–Ccr2*, *Il6–Il6ra/Il6st*, and *Tnf–Tnfrsf1a* signaling, mast cell-associated immune activation, and epithelial barrier gene expression. Resveratrol also altered gut microbial β-diversity, community composition, ecological assembly, and predicted functions.

**Conclusion:**

Resveratrol attenuates FD-like gastric dysmotility and duodenal inflammatory injury, potentially by reducing mast cell-associated mucosal immune activation, modulating chemokine- and cytokine-related immune–epithelial signaling, supporting epithelial barrier homeostasis, and reshaping gut microbiota.

## Introduction

Functional dyspepsia (FD) represents a common functional gastrointestinal disorder, typically presenting with postprandial fullness, early satiety, epigastric pain, and epigastric burning (1, 2). Since no clear organic lesions are usually detected in patients with FD, earlier studies mainly attributed its pathogenesis to abnormalities in gastric function, such as impaired gastric motility, delayed gastric emptying, defective gastric accommodation, and visceral hypersensitivity (3, 4). However, gastric dysfunction does not fully explain FD. Recent studies suggest that the duodenum also plays an important role. In patients with FD, several duodenal changes have been reported. These include impaired mucosal barrier function, low-grade inflammation, mucosal immune activation, and alterations in the gastroduodenal microbiota (5–7). This view is biologically plausible. After gastric emptying, as the first portion of the small intestine, the duodenum is directly exposed to substances within the intestinal lumen. It directly encounters gastric acid, bile acids, nutrients, microbial metabolites, and other factors. Therefore, an intact duodenal mucosa is important for normal gastrointestinal function. Stable local immune homeostasis is also needed (8–10). Based on these findings, the duodenal mucosal microenvironment may be a useful focus for understanding the mechanisms of FD (11).

Changes in duodenal mucosal immunity are now considered an important part of FD pathophysiology. In patients with FD, previous studies have reported impaired duodenal barrier integrity. Inflammatory cell infiltration and local immune activation have also been reported in FD (12). In the duodenal mucosa, immune cells and epithelial cells are closely linked. They do not work as separate parts. Instead, they may communicate through cytokines, chemokines, antigen-related signals, and other immune mediators. This communication may affect the epithelial barrier, mucosal repair, nerve sensitivity, and gastrointestinal motility.

Mast cells are important in this process. They are key cells in the mucosal immune system. Mast cells can release histamine, proteases, cytokines, chemokines, and other mediators. These substances may affect the epithelial barrier, nerve sensitivity, and gastrointestinal motility (13, 14). If mast cells build up or become overactive in the duodenal mucosa, they may promote low-grade inflammation and FD-related symptoms. This may occur through neuroimmune and immune–epithelial interactions (12, 15, 16). These findings suggest that reducing duodenal immune activation may help improve gastrointestinal dysfunction in FD. Mast cell-related inflammation and its link with epithelial homeostasis may be especially important (17).

Gut microbiota changes may also contribute to functional gastrointestinal disorders. The gut microbiota helps metabolize carbohydrates, amino acids, lipids, bile acids, and other substances. It also communicates with the host mucosa through short-chain fatty acids, microbial-associated molecular patterns, and other microbial metabolites. These products help regulate epithelial barrier function and immune responses (18–20).

Dysbiosis may disturb the luminal metabolic environment. It can also impair barrier integrity and alter mucosal immune responses. These changes may eventually affect gastrointestinal motility and visceral sensitivity (21). In this context, the gut microbiota may both influence and respond to mucosal immune dysregulation. It may help connect microbial changes with immune activation and epithelial barrier dysfunction.

Resveratrol is a naturally occurring dietary polyphenol. It is found in many plant-derived foods, including grapes, peanuts, and berries. It is also present in traditional medicinal plants. *Polygonum cuspidatum* is one important source of resveratrol and its glycoside derivatives (22–24). For this reason, resveratrol is often considered both a food-derived bioactive compound and a component of traditional botanical medicines (25, 26).

In recent years, resveratrol has received increasing attention in gastrointestinal research. This interest is partly related to its antioxidant and anti-inflammatory effects. Studies have also examined its effects on gut microbiota and intestinal barrier function (26–29). Compared with conventional drugs, dietary polyphenols often have broader but milder biological effects, which may make them suitable for long-term nutritional intervention. They may help maintain host balance by regulating inflammation, oxidative stress, epithelial barrier function, and the intestinal microecological environment (30–32).

Resveratrol and the gut microbiota appear to interact bidirectionally (27). On the one hand, intestinal microbes participate in the metabolic transformation of resveratrol, influencing its bioavailability and biological activity (33). On the other hand, resveratrol can reshape the composition and functional potential of the gut microbiota, with downstream effects on intestinal barrier function and immune homeostasis (34). From this perspective, resveratrol represents a useful candidate for investigating how dietary immunomodulatory compounds may regulate the mucosal immune–epithelial–microbial interface in FD-like pathology.

Although existing studies have shown that resveratrol has anti-inflammatory, barrier-protective, and microbiota-modulating effects, whether it can improve FD-like pathology through regulation of the duodenal mucosal microenvironment remains unclear. In particular, it is not known whether resveratrol can reshape mucosal immune signaling, influence candidate chemokine- and cytokine-related ligand–receptor signaling, and restore epithelial homeostasis and gut microbial balance in the context of FD. To address these questions, the present study established an FD-like mouse model and evaluated the effects of resveratrol on gastric emptying, duodenal histomorphology, mast cell accumulation, duodenal mucosal transcriptomic profiles, immune pathway enrichment, qPCR-validated mucosal homeostasis- and immune–epithelial signaling-related genes, and gut microbiota structure. By integrating histological, transcriptomic, qPCR, and microbiota analyses, this study aimed to determine whether resveratrol could alleviate FD-like pathology through coordinated regulation of duodenal mucosal immune activation, candidate immune–epithelial signaling axes, epithelial homeostasis, and the gut microbiota.

## 2 Materials and methods

### 2.1 Animals, FD model establishment, and resveratrol treatment

After acclimatization, male C57BL/6 mice were randomly assigned to the control, FD model, or resveratrol-treated group. The animals were 10 weeks old, with a body weight of 30 ± 2 g. They were kept in standard laboratory housing under a 12-hour light/dark cycle. Food and water were provided freely, except during the planned fasting periods. Approval for all animal-related procedures was obtained from the Animal Ethics Committee of Guangzhou University of Chinese Medicine.

The FD model was induced over 8 days using a multifactorial protocol adapted from previously reported methods, with modifications (35, 36). Mice in the FD and resveratrol groups received 0.2 mL of 0.1% iodoacetamide sucrose solution by gavage daily for 5 days, followed by 20-min forced swimming at 22 ± 1 °C and L-arginine gavage (5.7 g/kg) 1 h later. From days 6 to 8, cold rhubarb decoction (2.9 g/kg) was given by gavage. Alternate-day fasting/refeeding was applied during the modeling period. control mice received 0.2 mL of 2% sucrose daily.

After modeling, FD-like phenotypes were assessed, including general appearance, food intake, and gastric emptying. The resveratrol group received 100 mg/kg resveratrol by gavage once daily for 28 days, while control and FD mice received vehicle. After the treatment period, the mice were anesthetized using isoflurane and then euthanized by cervical dislocation. Duodenal tissues and duodenal contents were collected from the experimental mice and used for histology, RNA-seq, qPCR, and 16S rRNA sequencing.

### 2.2 Quantitative real-time PCR

Total RNA was isolated from duodenal tissues using TRIzol reagent (Invitrogen) and reverse-transcribed into cDNA with a Fermentas reverse transcription kit according to the manufacturer’s instructions. qRT-PCR was performed using SYBR Green PCR reagents (Thermo) on an ABI 7300 Real-Time PCR System. The PCR program consisted of 95 °C for 10 min, followed by 40 cycles of 95 °C for 15 s and 55 °C for 45 s. Melting curves were examined to confirm specific amplification.

Relative mRNA expression was calculated by the 2^−ΔΔCt method, with Actb used as the reference gene. The target genes included *Cyp24a1*, *Has2*, *Slc34a2*, *Ccl2*, *Ccr2*, *Il6*, *Il6ra*, *Il6st*, *Tnf*, *Tnfrsf1a*, *Kit*, *Mcpt1*, *Cpa3*, *Tjp1*, *Ocln, Il17a*, *Nfkbia*, *Cxcl10*, *Cd3e*, *Cd19*, *H2-Aa*, *Cldn1* and *Muc2*. The primer sequences used for qPCR are listed in **Supplementary Table 1**.

### 2.3 Histopathological assessment and toluidine blue staining

#### 2.3.1 H&E staining and histological scoring

Duodenal tissues were fixed in formalin for 48 h, routinely processed, embedded in paraffin, and cut into 4–7 μm sections. For histological assessment, the sections were stained with hematoxylin and eosin (H&E), dehydrated, cleared, and mounted with neutral resin.

Duodenal injury was evaluated according to villus integrity, crypt morphology, goblet cell changes, submucosal edema, and inflammatory cell infiltration. Each parameter was scored on a scale of 0–3, where 0 indicated no abnormality, 1 indicated mild changes, 2 indicated moderate changes, and 3 indicated severe changes. The scores for the five parameters were summed to obtain a total histological injury score ranging from 0 to 15.

For each animal, five randomly selected non-overlapping fields were examined. All sections were coded before evaluation. Histological scoring was independently performed by three experienced pathologists who were blinded to group allocation, and the mean of their scores was used for statistical analysis. Detailed scoring criteria are provided in **Supplementary Table 2**.

#### 2.3.2 Toluidine blue staining and mast cell counting

Additional sections were stained with toluidine blue to identify mast cells in the duodenal mucosa and were then differentiated in acetic acid solution. Mast cells were identified based on their metachromatic cytoplasmic granules.

For each animal, mast cells were counted in five randomly selected non-overlapping fields. All sections were coded before evaluation. Mast cell counting was independently performed by three experienced pathologists who were blinded to group allocation, and the mean count obtained by the three observers was used for statistical analysis.

All sections were examined under an Olympus CX41 microscope, and representative images were captured with a Nikon D5100 digital camera.

### 2.4 Sequencing and bioinformatic analyses

#### 2.4.1 RNA sequencing and transcriptomic analysis

Total RNA was extracted from duodenal mucosal samples using TRIzol^®^ Reagent and treated with DNase I to remove residual genomic DNA. RNA quality was checked with an Agilent 2100 Bioanalyzer, and RNA concentration was measured with a NanoDrop spectrophotometer. Qualified RNA samples were used for library preparation with the TruSeq RNA Sample Preparation Kit, followed by paired-end sequencing on an Illumina NovaSeq 6000 platform.

Raw reads were filtered to remove adaptor-contaminated reads, low-quality reads, and reads with ambiguous bases. The clean reads were then mapped to the reference genome using HISAT2, and gene-level counts were generated with HTSeq. Differential expression analysis was performed with edgeR. Genes with |log2 fold change| ≥ 1 and FDR < 0.05 were considered differentially expressed.

GO and KEGG enrichment analyses were used to assess functional and pathway changes. Upregulated and downregulated genes were analyzed separately when appropriate. Protein–protein interaction analysis was conducted to examine possible associations among DEGs. Four biological replicates were included in both the FD and resveratrol-treated groups.

#### 2.4.2 16S rRNA gene sequencing and gut microbiota analysis

Intestinal content samples were collected from control, FD model, and resveratrol-treated mice and stored at −80 °C. Microbial genomic DNA was extracted using the E.Z.N.A.^®^ Stool DNA Kit. Qualified DNA samples were used to amplify the bacterial V4–V5 region with the 515F/907R primers. The PCR products were purified, quantified, mixed at equal molar ratios, and subjected to paired-end high-throughput sequencing.

After sequencing, raw reads were assigned to their corresponding samples and filtered for quality. Low-quality reads, sequences with ambiguous bases, and reads with mismatched barcodes or primers were removed. Filtered paired-end reads were then merged. OTUs were clustered at 97% sequence similarity using UPARSE, and chimeric sequences were removed with UCHIME. Representative OTU sequences were annotated against the SILVA 16S rRNA database.

The final OTU table was used to analyze microbial diversity and community structure. β-diversity was assessed using Bray–Curtis distances and shown by principal coordinate analysis. PERMANOVA was used to compare community composition among groups. Shared and group-specific OTUs were identified by Venn analysis. Microbial composition was also compared at different taxonomic levels. Statistical significance was set at P < 0.05. Each group included four biological replicates.

### 2.5 Statistical analysis

GraphPad Prism 10.1.2 was used for statistical analysis, and data are presented as the mean ± SEM. Each dataset was first examined for normality and homogeneity of variance. For comparisons among more than two groups, normally distributed data with equal variance were analyzed by one-way ANOVA, followed by Tukey’s *post hoc* test. If these assumptions were not met, an appropriate non-parametric test was selected. When two groups were compared, an unpaired Student’s t-test was used where suitable. RNA-seq and 16S rRNA sequencing datasets were analyzed as described in the relevant methodological sections. A P value below 0.05 was considered statistically significant.

## 3 Results

### 3.1 Resveratrol improves FD-like gastric dysmotility in mice

**Figure 1:**
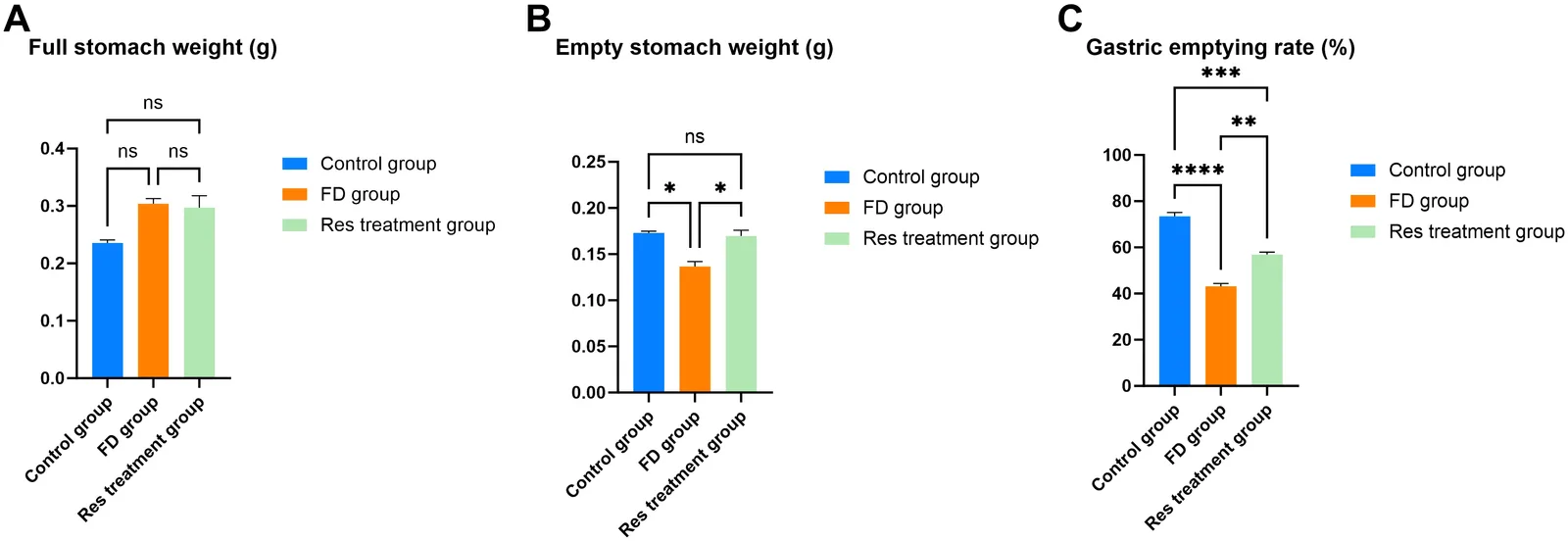
Resveratrol improves gastric emptying function in FD mice. Full stomach weight, empty stomach weight, and gastric emptying rate were measured to evaluate the establishment of the FD mouse model and the effect of resveratrol on gastric motility. **(A)** Full stomach weight; **(B)** empty stomach weight; **(C)** gastric emptying rate. Data are presented as the mean ± SEM (n = 12 mice per group from three independent experiments, with four mice per group in each experiment). ns, not significant; *P < 0.05, **P < 0.01, ***P < 0.001, ****P < 0.0001.

Full stomach weight, empty stomach weight, and gastric emptying rate were measured to validate the FD-like model and examine the effect of resveratrol on gastric motility. Full stomach weight was similar among the control, FD, and resveratrol-treated groups. A slight increase was observed in the FD group compared with the control group, but the difference was not significant (P > 0.05, **Figure 1A**). Resveratrol treatment also did not markedly change full stomach weight compared with the FD group. These results suggest that this parameter was not clearly affected by FD modeling or resveratrol intervention.

In contrast, empty stomach weight was significantly lower in the FD group than in the control group (P < 0.05, **Figure 1B**). This result indicates impaired gastric motility in FD mice. Consistently, the gastric emptying rate was also significantly reduced in the FD group compared with the control group (P < 0.0001, **Figure 1C**). These findings suggest delayed gastric emptying and support the successful induction of FD-like symptoms in mice.

After resveratrol treatment, empty stomach weight increased significantly relative to the FD group (P < 0.05). No significant difference was observed between the resveratrol-treated group and the control group (P > 0.05). These results suggest that resveratrol attenuated the FD-related reduction in empty stomach weight.

The gastric emptying rate showed a similar pattern. Compared with the control group, FD model mice showed a pronounced decrease in gastric emptying rate (P < 0.0001, **Figure 1C**), whereas resveratrol treatment significantly increased gastric emptying compared with the FD group (P < 0.01). However, the value did not fully return to the control level and remained significantly lower than that of the control group (P < 0.001). These findings support the successful establishment of the FD-like mouse model. They also suggest that resveratrol partly relieved delayed gastric emptying.

### 3.2 Resveratrol attenuates duodenal epithelial injury and inflammatory infiltration in FD mice

The gastric emptying results showed clear impairment of gastric motility in FD mice. Duodenal mucosal abnormalities may further contribute to FD-related gastrointestinal dysfunction. To examine this possibility, H&E staining was performed to assess duodenal tissue morphology.

**Figure 2:**
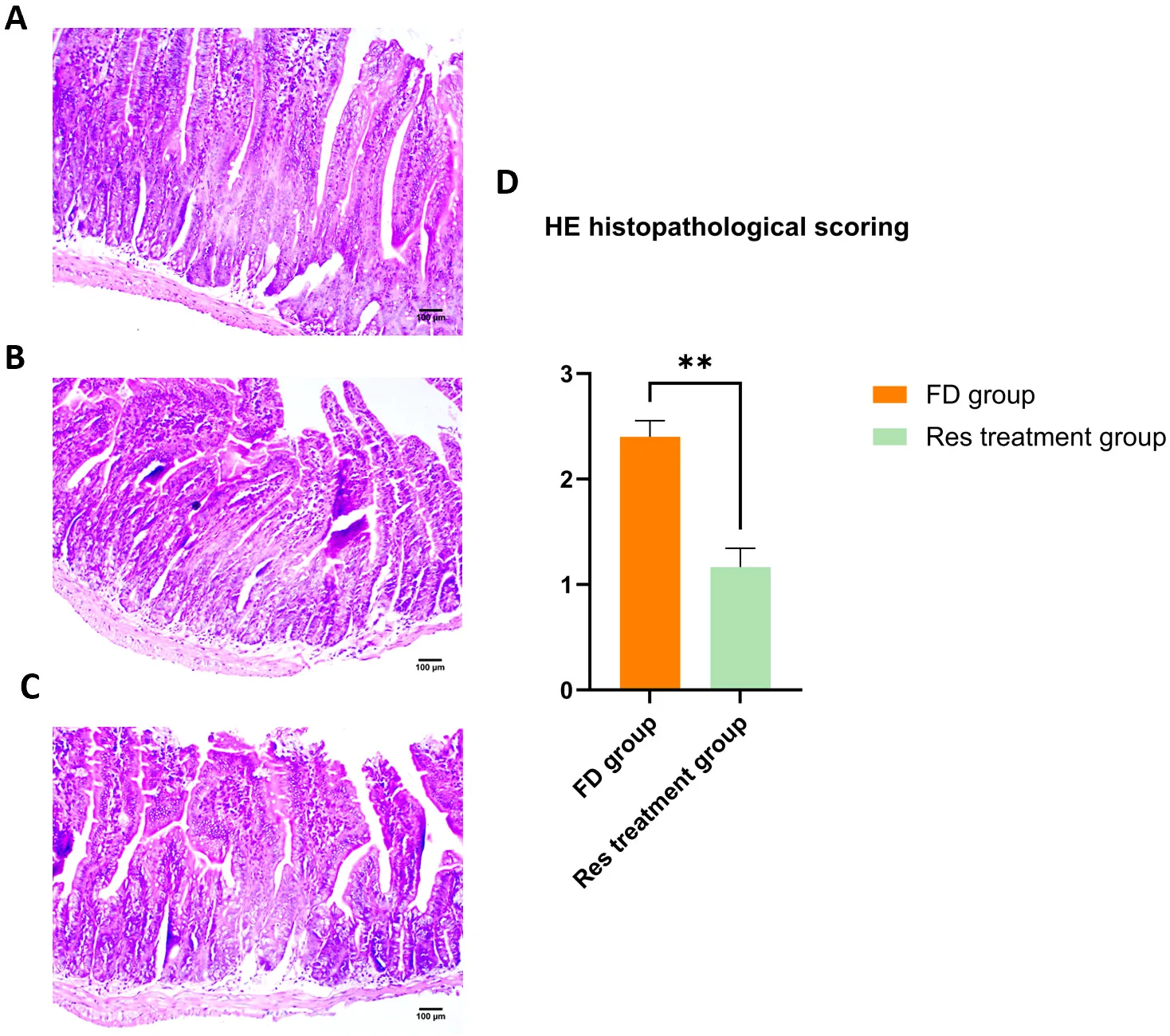
Resveratrol ameliorates duodenal mucosal injury in FD mice. Duodenal tissue morphology was assessed by H&E staining. **(A)** Control group: intact mucosal architecture, regularly arranged villi, normal crypt depth, and no obvious inflammatory cell infiltration. **(B)** FD group: disrupted mucosal glands and crypt structures, increased goblet cells, and inflammatory cell infiltration. **(C)** Res treatment group: no obvious abnormalities in mucosal glands or crypt structures, with only mild inflammatory cell infiltration in the submucosa. **(D)** HE histopathological score. Data are presented as the mean ± SEM (n = 12 mice per group from three independent experiments, with four mice per group in each experiment). Each biological sample was measured in technical triplicate, and the mean of the three technical replicates was used for statistical analysis. **P < 0.01. Scale bar = 100 μm.

The control group showed well-preserved duodenal mucosal architecture.The duodenal tissue showed a clear and well-organized structure. The villi were slender and neatly aligned. No clear villus collapse, detachment, breakage, or atrophy was observed. The crypts were orderly and remained at a normal depth (**Figure 2A**).

Histological examination revealed obvious changes in the duodenum of FD model mice. Compared with the control group, the FD group had disorganized mucosal glands and crypts. An increase in goblet cells was also observed. Obvious inflammatory cell infiltration was also observed (**Figure 2B**). These changes suggest that the FD model induced duodenal mucosal injury and local inflammation.

After resveratrol intervention, duodenal mucosal injury was noticeably reduced. The resveratrol-treated mice showed relatively preserved glandular and crypt structures. Only mild submucosal inflammatory infiltration was observed (**Figure 2C**). This improvement was also reflected in the histopathological scores. Resveratrol treatment led to a significant reduction in these scores relative to the FD group (P < 0.01, **Figure 2D**). Together, these findings indicate that resveratrol mitigates duodenal mucosal damage and local inflammation in FD mice.

### 3.3 Resveratrol reduces mast cell accumulation in the duodenal mucosa of FD mice

Resveratrol treatment improved duodenal mucosal morphology and local inflammation. We therefore performed toluidine blue staining to further examine mast cell infiltration in the duodenal mucosa.

**Figure 3:**
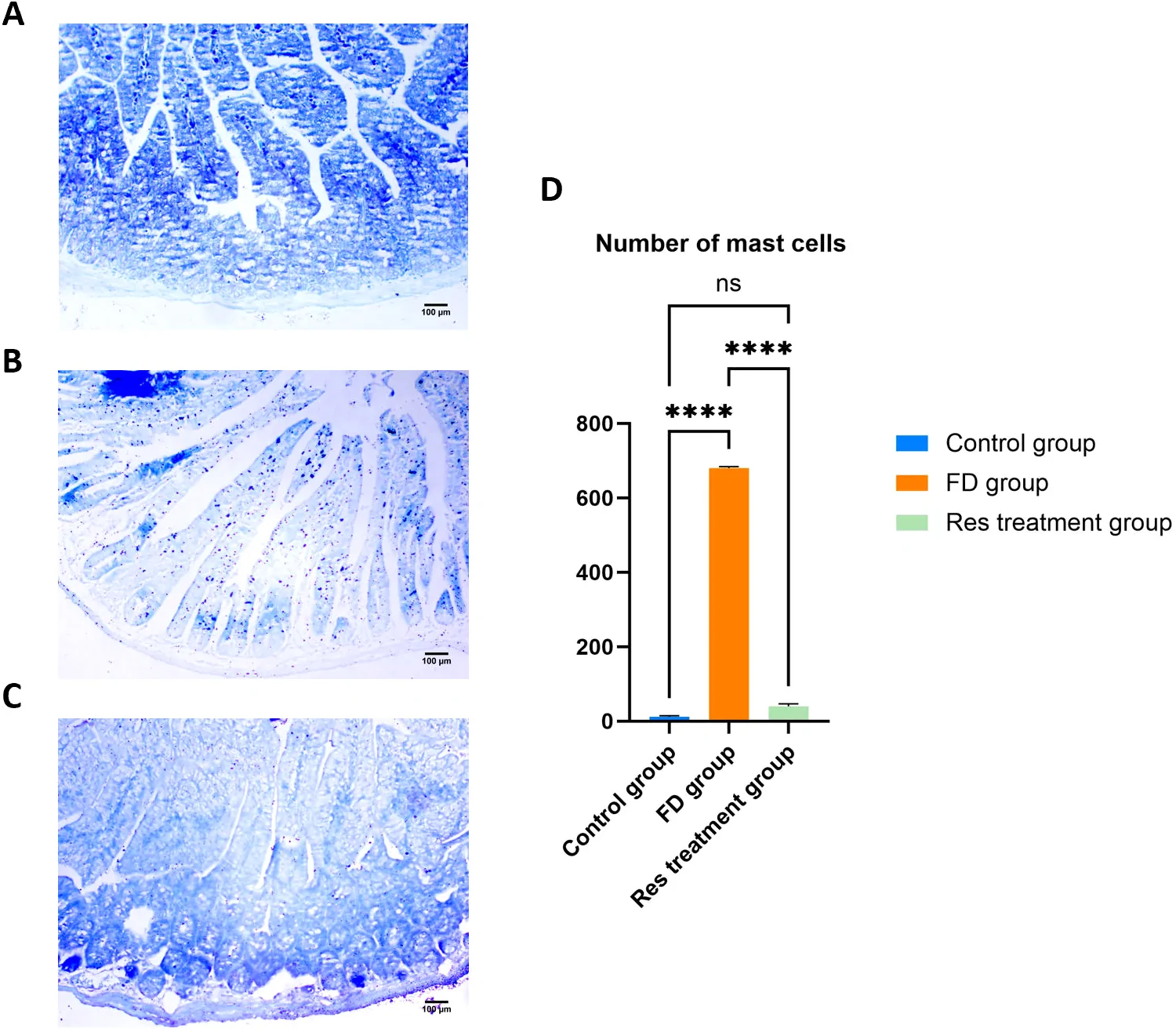
Resveratrol reduces duodenal mucosal mast cell infiltration in FD mice. Mast cells in the duodenal mucosa were assessed by toluidine blue staining. **(A)** Representative image of the Control group, showing few mast cells. **(B)** Representative image of the FD group, showing increased mast cell infiltration. **(C)** Representative image of the Res treatment group, showing reduced mast cell infiltration after resveratrol treatment. **(D)** Quantification of mast cell numbers. Data are presented as the mean ± SEM (n = 12 mice per group from three independent experiments, with four mice per group in each experiment). ****P < 0.0001; ns, not significant. Scale bar = 100 μm.

Representative staining showed few mast cells in the duodenal mucosa of control mice(**Figure 3A**); in the FD group, mast cells were markedly increased (**Figure 3B**), suggesting mucosal immune activation; following resveratrol treatment, mast cell numbers were markedly reduced (**Figure 3C**). Quantification revealed that mast cell numbers were markedly higher in the FD group than in the control group (P < 0.0001). Resveratrol treatment significantly lowered this increase compared with the FD group (P < 0.0001), bringing mast cell numbers to a level similar to that observed in control mice (P > 0.05, **Figure 3D**).These results indicate that resveratrol attenuates mucosal immune activation in FD mice, as reflected by reduced mast cell infiltration in the duodenal mucosa.

### 3.4 Resveratrol reshapes immune-related transcriptional programs in the duodenal mucosal microenvironment

#### 3.4.1 Resveratrol modulates mucosal immune defense, cytokine signaling, and adaptive immune pathways

The toluidine blue staining results mentioned above showed increased mast cell accumulation in the duodenal mucosa of FD mice, indicating the involvement of mucosal immune activation in FD-associated duodenal abnormalities. We next performed RNA sequencing of duodenal mucosal samples to investigate the molecular changes that may underlie the effect of resveratrol on this immune-related alteration.

**Figure 4:**
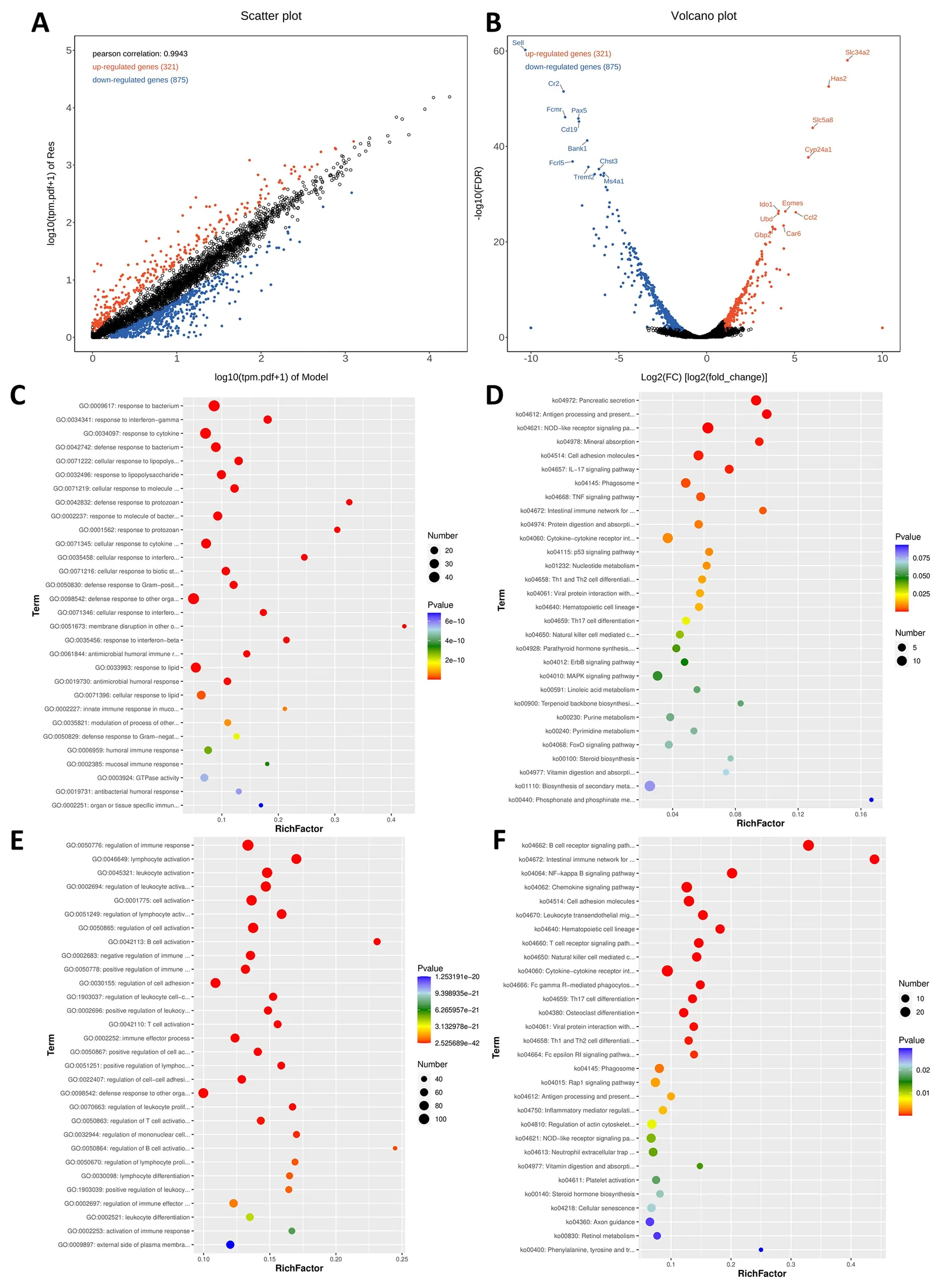
Resveratrol remodels immune-related transcriptional networks in the duodenal mucosal microenvironment of FD mice. RNA-seq was performed to characterize transcriptional changes related to mucosal immune regulation and immune–epithelial signaling after resveratrol treatment (n = 4 mice per group). **(A)** Scatter plot showing gene expression correlation between the FD and Res treatment groups. **(B)** Volcano plot showing differentially expressed genes between the FD and Res treatment groups. **(C)** GO enrichment analysis of upregulated DEGs. **(D)** KEGG enrichment analysis of upregulated DEGs. **(E)** GO enrichment analysis of downregulated DEGs. **(F)** KEGG enrichment analysis of downregulated DEGs.

Although the FD and resveratrol-treated groups showed broadly similar transcriptomic profiles, the Pearson correlation coefficient remained high at 0.9943 (**Figure 4A**). Differential expression analysis identified 1,196 DEGs following resveratrol treatment compared with the FD group, including 321 upregulated and 875 downregulated genes (**Figure 4B**). Genes such as Slc34a2, Has2, Slc5a8, and Cyp24a1 were upregulated, whereas Sell, Cr2, Fcmr, Pax5, and Cd19 were markedly downregulated. These findings suggest that resveratrol induces substantial but selective transcriptional changes in the duodenal mucosa of FD mice, involving genes associated with mucosal immunity and epithelial homeostasis.

GO enrichment analysis indicated that the upregulated DEGs were involved in immune defense and mucosal immunity, including responses to bacteria, cytokines, and lipopolysaccharide, as well as antibacterial defense, mucosal immune responses, and humoral immune responses (**Figure 4C**). KEGG analysis further associated the upregulated genes with antigen processing and presentation, NOD-like receptor signaling, cell adhesion molecules, IL-17 and TNF signaling, phagosome, the intestinal immune network for IgA production, cytokine–cytokine receptor interactions, and Th1/Th2/Th17 cell differentiation (**Figure 4D**).

The downregulated DEGs were enriched in GO terms related to the regulation of immune responses, activation of lymphocytes, leukocytes, B cells, and T cells, and immune effector processes (**Figure 4E**). KEGG analysis associated these genes with B-cell and T-cell receptor signaling, NF-κB and chemokine signaling, cell adhesion molecules, leukocyte transendothelial migration, hematopoietic cell lineage, natural killer cell-mediated cytotoxicity, cytokine–cytokine receptor interactions, Fc gamma R-mediated phagocytosis, Th17 cell differentiation, phagosome, and NOD-like receptor signaling (**Figure 4F**).

Overall, the GO and KEGG analyses indicate that resveratrol treatment alters transcriptional networks associated with immune responses, cytokine signaling, mucosal defense, and epithelial function in the duodenal mucosa of FD mice.

#### 3.4.2 GO-DAG analysis reveals hierarchical immune and cytokine-related functional networks

GO-DAG analysis was further performed to clarify the hierarchical relationships among enriched GO terms for the upregulated and downregulated DEGs across the BP, CC, and MF categories. In the BP-DAG analysis of the downregulated genes, enriched terms included regulation of immune response, regulation of cell, leukocyte, and lymphocyte activation, as well as cell, leukocyte, and lymphocyte activation (**Supplementary Figure 1A**). The CC-DAG highlighted the cell surface, receptor complexes, the external side of the plasma membrane, and the immunological synapse (**Supplementary Figure 1B**). The MF-DAG included CD4 receptor binding, cytokine receptor activity, phosphoprotein binding, and other functions related to the binding of phosphorylated proteins or amino acid residues (**Supplementary Figure 1C**).

The BP-DAG analysis of the upregulated genes highlighted host-defense-related processes, including responses to bacteria, cytokines, and lipopolysaccharide, together with defense responses, innate immune responses, and humoral immune responses (**Supplementary Figure 2A**). Enriched CC terms included the extracellular region, the external side of the plasma membrane, and MHC protein complexes (**Supplementary Figure 2B**). In the MF-DAG analysis, the enriched terms were mainly associated with cytokine- and chemokine-related functions, including cytokine activity, receptor ligand activity, cytokine receptor binding, chemokine activity, chemokine receptor binding, and MHC protein complex binding (**Supplementary Figure 2C**).

Collectively, these findings are consistent with the involvement of the resveratrol-responsive DEGs in immune cell activation, cytokine- and chemokine-related functions, antigen-related processes, and mucosal defense responses.

#### 3.4.3 PPI network identifies immune and epithelial homeostasis-related hub genes

**Figure 5:**
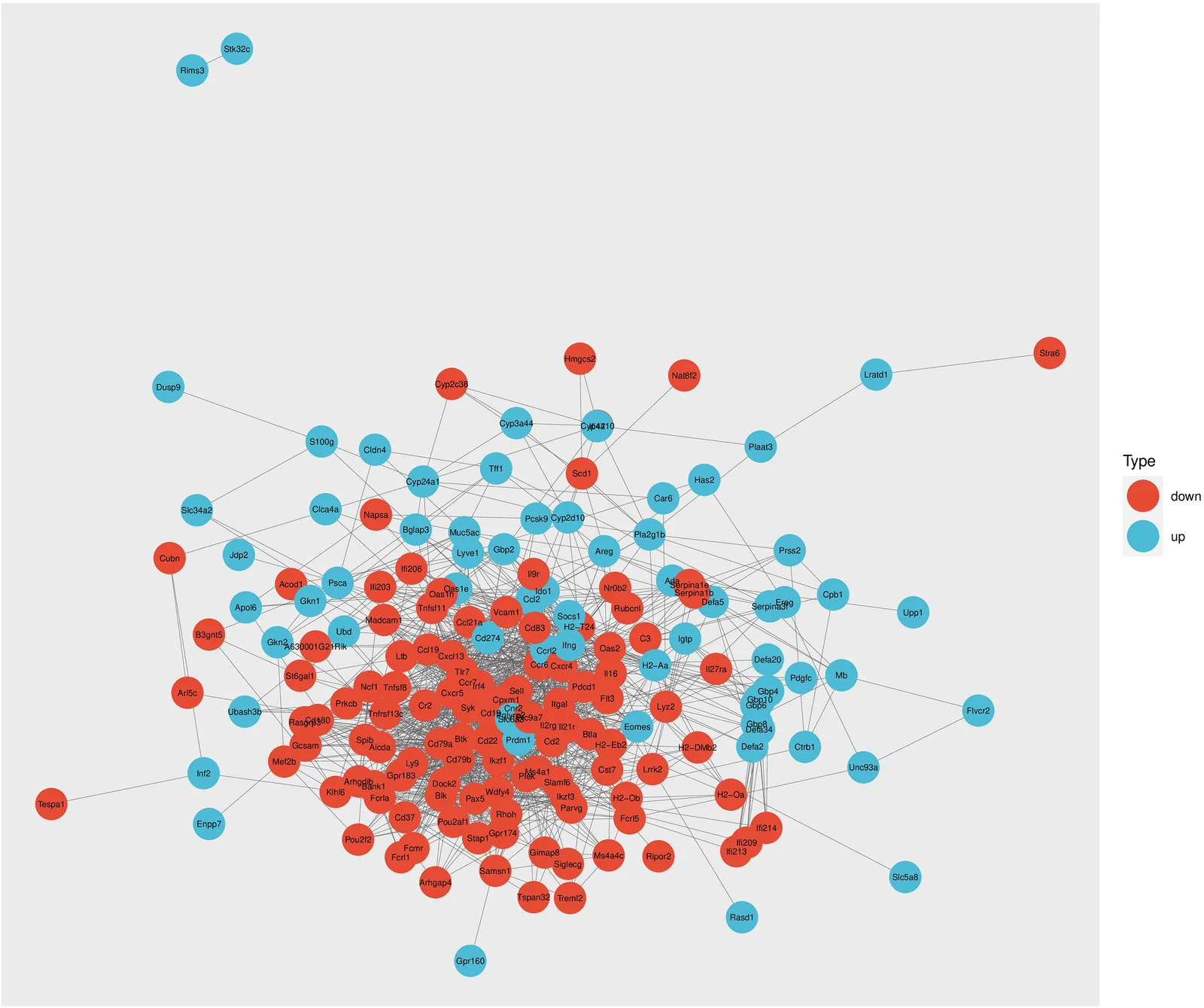
PPI network of DEGs between the FD and Res treatment groups. The protein–protein interaction network was constructed using differentially expressed genes identified from RNA-seq analysis (n = 4 mice per group). Nodes represent proteins encoded by DEGs, and edges indicate predicted protein–protein interactions.

A PPI network was further generated to explore potential functional associations among the DEGs. As shown in **Figure 5**, the network revealed extensive interactions involving both upregulated and downregulated genes after resveratrol treatment. Notably, several immune- and inflammation-associated genes, including *Ccl2*, *Il6*, *Ccr2*, *Cd274*, *Tlr7*, *Cd19*, *Pax5*, *Cr2*, and *Sell*, appeared as key nodes, indicating their possible involvement in the regulation of mucosal immune responses in FD mice. The network also contained epithelial stability- and transport-related genes, including *Slc34a2*, *Has2*, and *Slc5a8*. Together with the DEG, GO/KEGG, and GO-DAG analyses, this result suggests that resveratrol may affect duodenal mucosal gene expression in FD mice. These changes may involve immune responses, inflammation, and epithelial homeostasis.

### 3.5 Resveratrol enhances the expression of genes related to mucosal metabolism and epithelial function in the duodenal mucosa

Based on the RNA-seq results, *Cyp24a1*, *Has2*, and *Slc34a2* were selected for further analysis because of their potential relevance to duodenal mucosal function. *Cyp24a1* is involved in vitamin D metabolism and may be linked to mucosal immune regulation and epithelial homeostasis (37–40). *Has2* encodes hyaluronan synthase and may support mucosal barrier maintenance through extracellular matrix formation. *Slc34a2* is an intestinal phosphate transporter related to epithelial cell homeostasis and energy metabolism. These genes were therefore examined by qPCR to validate key RNA-seq changes and to explore the possible role of resveratrol in mucosal immunity and epithelial homeostasis.

**Figure 6:**
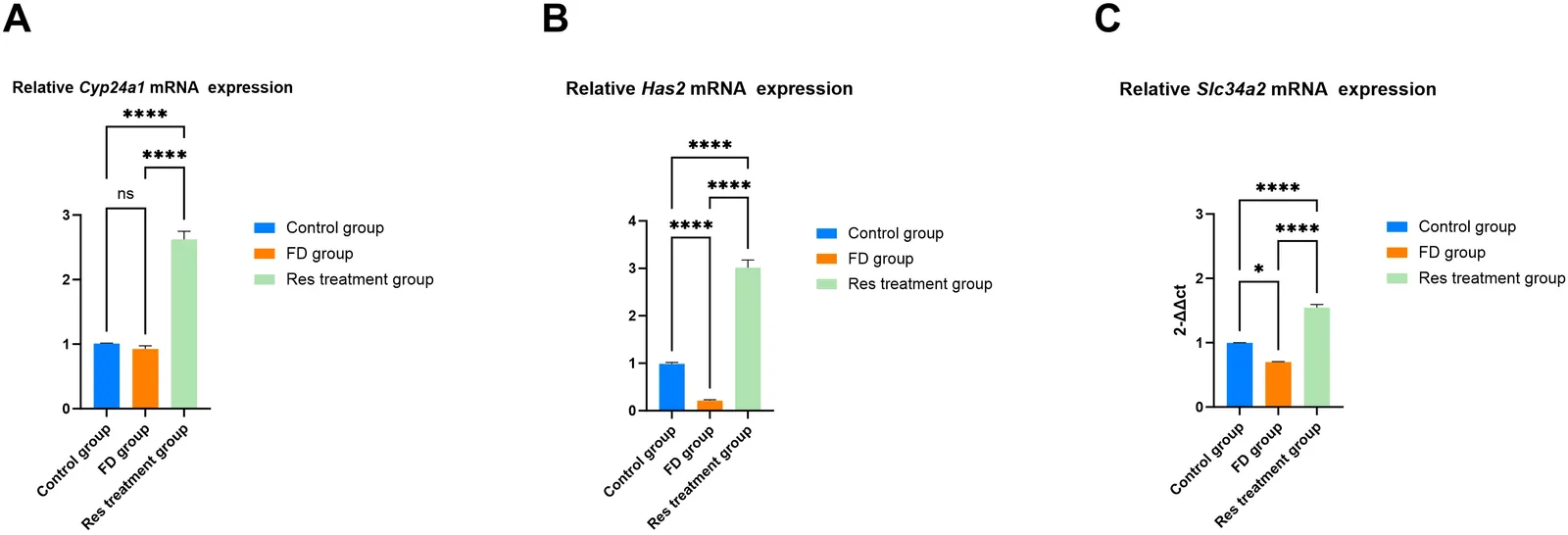
Resveratrol upregulates genes related to mucosal metabolism and epithelial function in the duodenal mucosa of FD mice. qPCR was performed to measure the expression levels of selected genes related to mucosal metabolism and epithelial function in the duodenal mucosa of each group. Relative expression levels were calculated using the 2^−ΔΔCt method. **(A)** *Cyp24a1* expression; **(B)** *Has2* expression; **(C)** *Slc34a2* expression. Data are presented as the mean ± SEM (n = 12 mice per group from three independent experiments, with four mice per group in each experiment). Each biological sample was measured in technical triplicate, and the mean of the three technical replicates was used for statistical analysis. *P < 0.05, ****P < 0.0001; ns, not significant.

Compared with the control group, the FD group showed no significant change in *Cyp24a1* expression (P > 0.05; **Figure 6A**), whereas Has2 expression was markedly decreased (P < 0.0001; **Figure 6B**). *Slc34a2* expression was also significantly reduced in the FD group (P < 0.05; **Figure 6C**). After resveratrol treatment, the expression of *Cyp24a1*, *Has2*, and *Slc34a2* was significantly increased compared with the FD group (all P < 0.0001; **Figures 6A–C**). The expression levels of all three genes were also significantly higher in the resveratrol-treated group than in the control group (all P < 0.0001; **Figures 6A–C**).

The qPCR results were consistent with the RNA-seq data. These findings indicate that resveratrol induced *Cyp24a1* expression and reversed the FD-associated reductions in *Has2* and *Slc34a2* expression. These transcriptional changes may be related to the regulation of vitamin D metabolism, extracellular matrix remodeling, and epithelial transport in the duodenal mucosa.

### 3.6 Resveratrol modulates candidate immune–parenchymal ligand–receptor-related axes in the duodenal mucosal microenvironment

The RNA-seq and PPI analyses suggested that resveratrol affected both immune-related signaling and epithelial homeostasis-related genes in the duodenal mucosa. To further define these changes within the local immune–parenchymal microenvironment, qPCR was used to examine representative chemokine- and cytokine-related ligand–receptor signaling genes, mast cell-associated markers, and epithelial barrier-related genes. In this study, the epithelial barrier compartment was considered the major parenchymal component of the duodenal mucosa, while mast cell-associated and cytokine/chemokine-related genes were used to reflect local immune activation.

**Figure 7:**
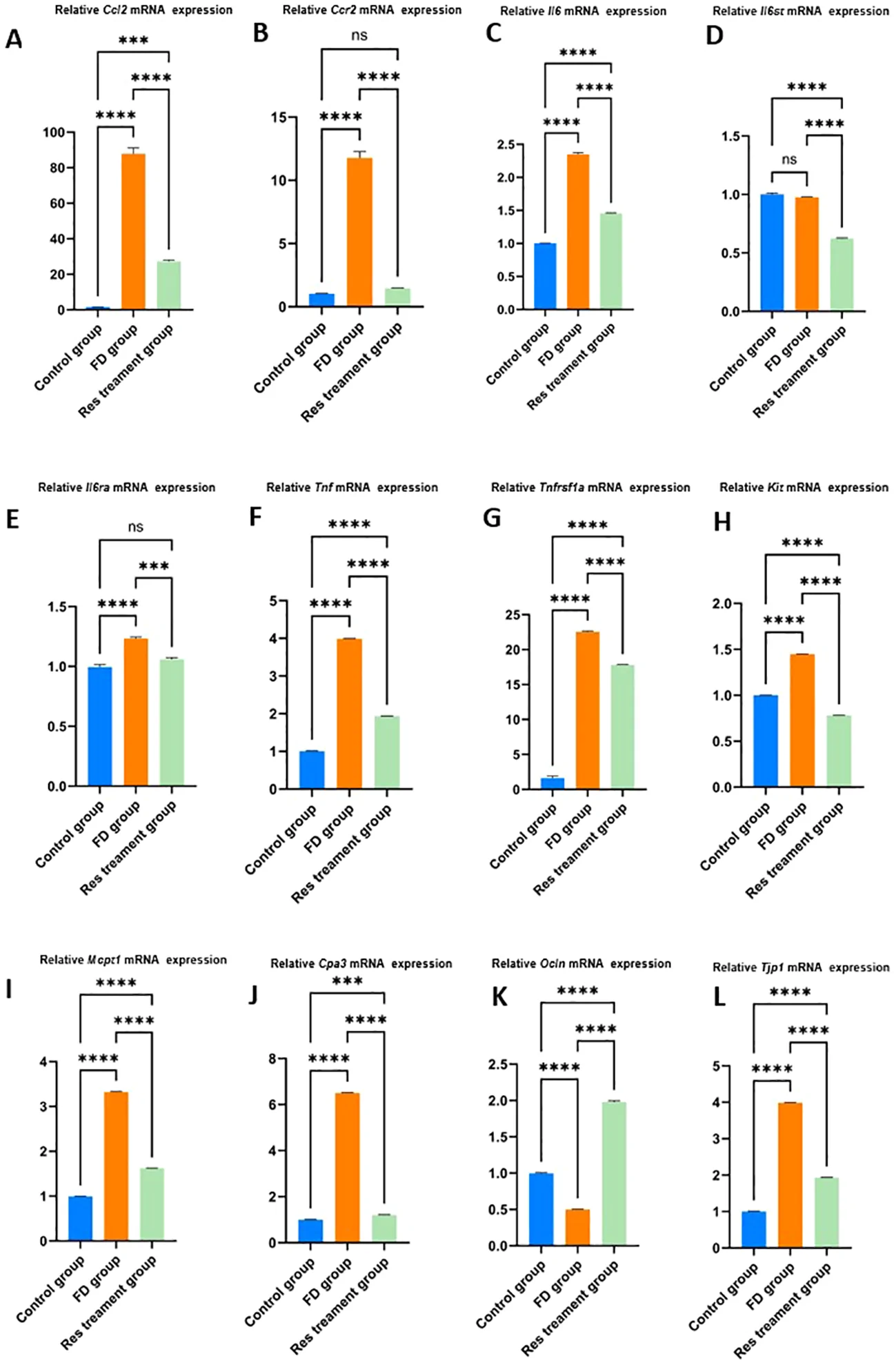
Resveratrol modulates immune–epithelial signaling-, mast cell-, and barrier-related gene expression in the duodenal mucosa of FD mice. qPCR was performed to measure the expression levels of representative chemokine- and cytokine-related signaling genes, mast cell-associated markers, and epithelial barrier-related genes in the duodenal mucosa of each group. Relative expression levels were calculated using the 2^−ΔΔCt method. **(A)** *Ccl2* expression; **(B)** *Ccr2* expression; **(C)** *Il6* expression; **(D)** *Il6st* expression; **(E)** *Il6ra* expression; **(F)** *Tnf* expression; **(G)** *Tnfrsf1a* expression; **(H)** *Kit* expression; **(I)** *Mcpt1* expression; **(J)** *Cpa3* expression; **(K)** *Ocln* expression; **(L)** *Tjp1* expression. Data are presented as the mean ± SEM (n = 12 mice per group from three independent experiments, with four mice per group in each experiment). Each biological sample was measured in technical triplicate, and the mean of the three technical replicates was used for statistical analysis.***P < 0.001, ****P < 0.0001; ns, not significant.

Compared with the control group, FD mice showed marked increases in the duodenal expression of *Ccl2* and *Ccr2* (both P < 0.0001; **Figures 7A, B**). Resveratrol treatment significantly reduced both genes (both P < 0.0001). *Ccr2* returned to a level comparable to that in the control group, whereas Ccl2 remained modestly elevated (P < 0.001).

The components of the *Il6*-related signaling pathway showed distinct expression patterns (**Figures 7C–E**). Il6 and Il6ra were significantly upregulated in the FD group (both P < 0.0001) and decreased after resveratrol treatment (P < 0.0001 and P < 0.001, respectively). *Il6* remained above the control level, whereas *Il6ra* was restored to a comparable level. Il6st did not differ between the control and FD groups, but its expression was significantly reduced by resveratrol compared with both groups (both P < 0.0001). *Tnf* and *Tnfrsf1a* were also elevated in FD mice and reduced after treatment (all P < 0.0001; **Figures 7F, G**), although both remained higher than in the control group.

The mast cell-associated markers *Kit*, *Mcpt1*, and *Cpa3* were significantly increased in the FD group and reduced after resveratrol treatment (all P < 0.0001; **Figures 7H–J**), in agreement with the toluidine blue staining results. After treatment, *Kit* expression fell below the control level, whereas *Mcpt1* and *Cpa3* remained elevated (P < 0.0001 and P < 0.001, respectively).

*Ocln* and *Tjp1* showed different expression patterns (**Figures 7K, L**). *Ocln* was reduced in FD mice and markedly increased after resveratrol treatment, reaching a level higher than that in the control group (all P < 0.0001). By contrast, Tjp1 was elevated in the FD group and decreased following treatment (all P < 0.0001), but remained above the control level.

These findings show that resveratrol substantially altered chemokine-, cytokine-, mast cell-, and epithelial barrier-related gene expression in the duodenal mucosa. The extent of recovery varied among genes, with some returning to control levels and others showing partial responses.

### 3.7 Resveratrol reshapes broader mucosal immune and epithelial response programs in the duodenum

To further characterize the mucosal transcriptional changes associated with resveratrol treatment, we examined representative genes related to inflammation, chemokine responses, adaptive immunity, antigen presentation, and epithelial barrier function by qPCR.

**Figure 8:**
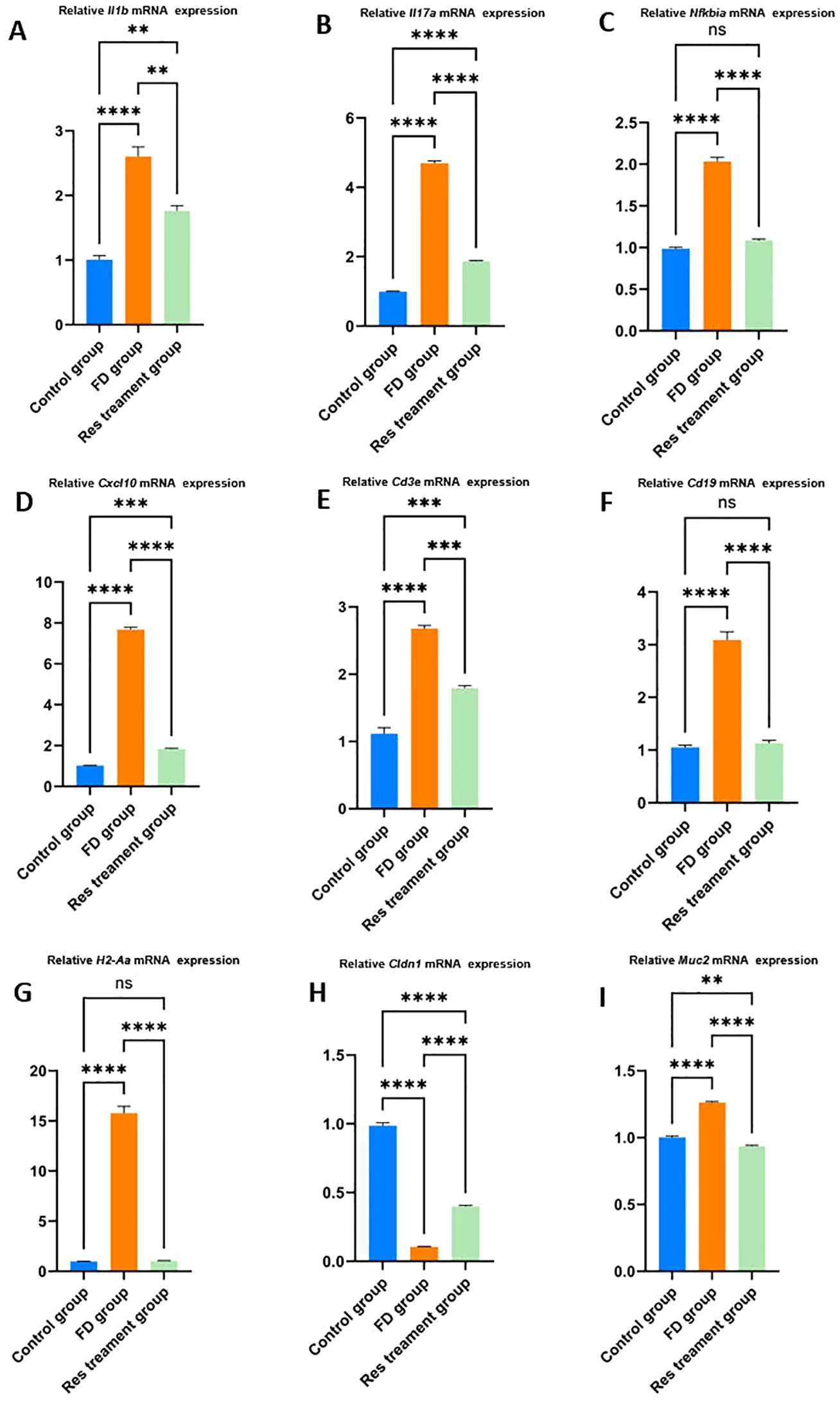
Resveratrol alters inflammatory, immune-related, and epithelial barrier-related gene expression in the duodenal mucosa of FD mice. qPCR was performed to measure the expression levels of representative inflammation- and chemokine-related genes, adaptive immune- and antigen presentation-related genes, and epithelial barrier-related genes in the duodenal mucosa of each group. Relative expression levels were calculated using the 2^−ΔΔCt method. **(A)** *Il1b* expression; **(B)** *Il17a* expression; **(C)** Nfkbia expression; **(D)** *Cxcl10* expression; **(E)** *Cd3e* expression; **(F)** *Cd19* expression; **(G)** *H2-Aa* expression; **(H)** *Cldn1* expression; **(I)** *Muc2* expression. Data are presented as the mean ± SEM (n = 12 mice per group from three independent experiments, with four mice per group in each experiment). Each biological sample was measured in technical triplicate, and the mean of the three technical replicates was used for statistical analysis. **P < 0.01, ***P < 0.001, ****P < 0.0001; ns, not significant.

Compared with the control group, the FD group showed markedly increased expression of Il1b, *Il17a*, *Nfkbia*, and *Cxcl10* in the duodenal mucosa (all P < 0.0001; **Figures 8A–D**). Resveratrol treatment significantly reduced the expression of Il1b (P < 0.01), *Il17a*, *Nfkbia*, and *Cxcl10* (all P < 0.0001) compared with the FD group. After treatment, Nfkbia expression was comparable to that in the control group (P > 0.05), whereas *Il1b*, *Il17a*, and *Cxcl10* remained significantly elevated (P < 0.01, P < 0.0001, and P < 0.001, respectively).

The expression of *Cd3e*, *Cd19*, and *H2-Aa* was also significantly higher in the FD group than in the control group (all P < 0.0001; **Figures 8E–G**). Resveratrol treatment reduced the expression of *Cd3e* (P < 0.001), *Cd19*, and *H2-Aa* (both P < 0.0001) relative to the FD group. *Cd19* and *H2-Aa* returned to levels comparable to those in the control group (both P > 0.05), while *Cd3e* remained significantly elevated (P < 0.001).

Cldn1 expression was markedly reduced in the FD group compared with the control group (P < 0.0001; **Figure 8H**). Although resveratrol significantly increased *Cldn1* expression relative to the FD group (P < 0.0001), its level remained lower than that in the control group (P < 0.0001). By contrast, *Muc2* expression was increased in the FD group (P < 0.0001; **Figure 8I**) and significantly decreased after resveratrol treatment (P < 0.0001). *Muc2* expression in the resveratrol-treated group was slightly lower than that in the Control group (P < 0.01).

Overall, resveratrol attenuated the inflammatory, immune-related, and epithelial barrier-associated transcriptional changes observed in the duodenal mucosa of FD mice. Some genes returned to levels comparable to those in the control group, whereas others showed only partial recovery.

### 3.8 Resveratrol is associated with changes in gut microbial diversity and community structure in FD mice

**Figure 9:**
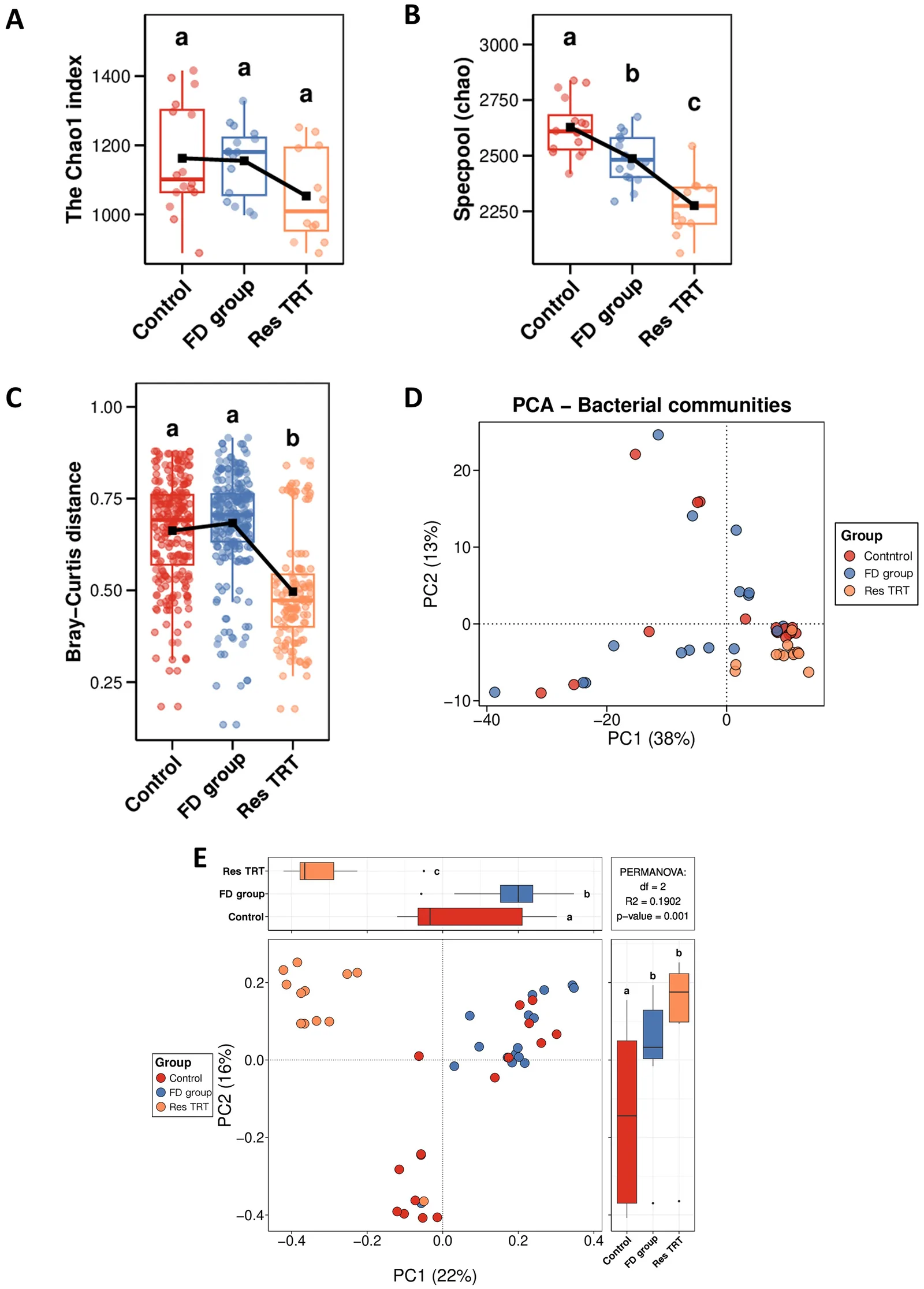
Resveratrol shifts gut microbiota diversity and community separation in FD mice. Gut microbiota diversity and community structure were analyzed across the Control, FD, and resveratrol-treated groups (n = 4 mice per group). **(A)** Chao1 index assessing species richness; **(B)** Specpool (Chao) index estimating community richness; **(C)** β-diversity analysis based on Bray–Curtis distance; **(D)** Principal component analysis (PCA) showing sample distribution along PC1 and PC2; **(E)** Principal coordinates analysis (PCoA) depicting differences in community structure based on Bray–Curtis distance, with PERMANOVA results. Adonis, ANOSIM, and MRPP analyses all indicated significant differences between groups. Data are presented as mean ± SEM.

To examine whether resveratrol was associated with changes in gut microbial diversity in FD mice, α-diversity was first compared among the groups. The Chao1 index showed no significant difference among the control, FD, and resveratrol-treated groups, suggesting that overall species richness was not markedly changed (**Figure 9A**).

In contrast, the Specpool (Chao) index differed significantly among groups. It was highest in the control group, decreased in the FD group, and declined further after resveratrol treatment (**Figure 8B**). This pattern suggests changes in estimated microbial richness and a possible shift in community composition.

Bray–Curtis-based β-diversity analysis showed relatively similar microbial communities in the control and FD groups. However, the resveratrol-treated group was clearly separated from both groups (**Figure 9C**).This result suggests that resveratrol had a pronounced effect on gut microbiota composition in FD mice. Consistently, PCA revealed an obvious separation of Res-treated samples from those of the control and FD groups along PC1 and PC2, explaining 38% and 13% of the total variation, respectively (**Figure 9D**). Most samples in the Res group were located on the positive side of PC1, further supporting the distinct microbial composition induced by resveratrol.

PCoA analysis further confirmed the separation among the three groups, with PC1 and PC2 explaining 22% and 16% of the variation, respectively (**Figure 9E**). PERMANOVA analysis demonstrated a significant effect of treatment on gut microbial composition (**Figure 9E**). Consistently, Adonis (R² = 0.1329, F = 3.1421, P = 0.001), ANOSIM (R = 0.2136, P = 0.001), and MRPP (A = 0.0544, P = 0.001) analyses all confirmed statistically significant differences among groups.

Taken together,α-diversity analysis showed differences in selected measures of microbial richness, whereas β-diversity analysis revealed clear differences in overall microbial community structure. In particular, the microbial composition of the resveratrol-treated group was distinct from those of both the control and FD groups.

### 3.9 Resveratrol treatment is associated with differences in gut microbial composition, ecological assembly, and predicted functional profiles in FD mice

**Figure 10:**
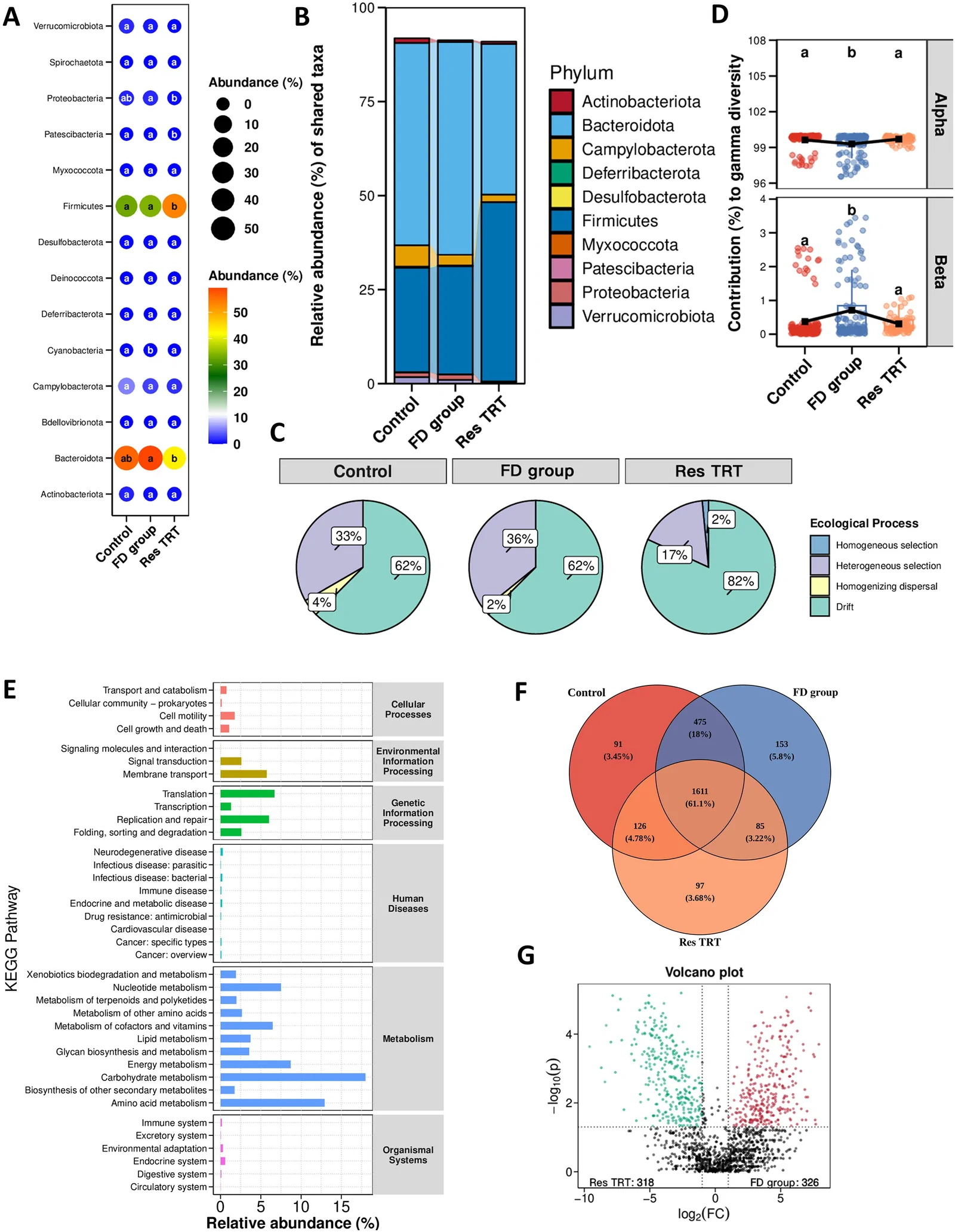
Resveratrol remodels the gut microbial ecosystem in FD mice. Gut microbiota composition, community assembly, diversity partitioning, predicted functions, and differentially abundant taxa were analyzed across the Control, FD, and resveratrol-treated groups (n = 4 mice per group). **(A)** Bubble plot showing relative abundance of dominant phyla; **(B)** stacked bar plot displaying phylum-level composition across groups; **(C)** contribution of ecological processes to community assembly; **(D)** Rao decomposition showing alpha and beta components of gamma diversity; **(E)** functional prediction analysis showing main functional categories; **(F)** Venn diagram illustrating shared and unique taxa among groups; **(G)** volcano plot of differentially abundant taxa between the FD and Res treatment groups. Data are presented as mean ± SEM.

Subsequently, we evaluated changes in gut microbiota composition at the phylum level. The bubble plot showed that Bacteroidota and Firmicutes were the dominant phyla across all groups, although their relative abundances differed significantly (**Figure 10A**). Compared with the FD group, the resveratrol-treated group showed a higher relative abundance of Firmicutes and a lower relative abundance of Bacteroidota. Proteobacteria and Patescibacteria also differed significantly between the groups. A similar pattern was observed in the stacked bar plot (**Figure 10B**). Together, these results suggest that resveratrol changed the phylum-level structure of the gut microbiota, mainly through Firmicutes expansion and Bacteroidota reduction.

Microbial community assembly was then examined to clarify whether resveratrol changed the ecological forces shaping the gut microbiota. Drift accounted for the largest proportion in the control and FD groups, both at 62%, and increased to 82% in the resveratrol-treated group. By contrast, heterogeneous selection decreased after resveratrol treatment, from 33% in controls and 36% in FD mice to 17% in the resveratrol-treated group (**Figure 10C**). These results indicate that resveratrol shifted gut microbiota assembly in FD mice toward a pattern more strongly influenced by stochastic drift and less by heterogeneous selection.

Rao decomposition analysis suggested that the balance between within-sample diversity and between-sample variation in shaping gamma diversity was not consistent across groups. The alpha component was significantly different in the FD group compared with both the control and Res groups, whereas the Res group remained close to the control group. The beta component showed a similar pattern, with a higher contribution in the FD group than in the control and Res groups, while the Res and control groups were not significantly different from each other (**Figure 10D**). These results show that the diversity contribution pattern in the resveratrol-treated group was closer to that observed in the control group than to that in the FD group.

Functional analysis using KEGG Level 2 pathways revealed that the functions of these three microbial groups are primarily distributed across the domains of metabolism, genetic information processing, environmental information processing, and cellular processes (**Figure 10E**). In the metabolism category, carbohydrate, amino acid, energy, nucleotide and lipid metabolism, as well as cofactor and vitamin metabolism, constitute the main functional groups. Pathways associated with membrane transport, signal transduction, translation, and replication and repair also accounted for a substantial proportion of the predicted functions. Collectively, the predicted microbial functions in both FD and resveratrol-treated mice were mainly related to nutrient utilization, energy metabolism, transport activities, and genetic information processing.

The Venn diagram showed that 1,611 taxa were shared among the control, FD, and resveratrol-treated groups, accounting for 61.1% of all detected taxa. This result indicates that a large proportion of the gut microbial community was common to all groups (**Figure 10F**). At the same time, each group also contained group-specific taxa, with 91 unique taxa in the control group, 153 in the FD group, and 97 in the resveratrol-treated group. Differential abundance analysis further showed a distinct distribution of taxa between the FD and resveratrol-treated groups, with 318 taxa enriched in the resveratrol-treated group and 326 taxa enriched in the FD group (**Figure 10G**). These results suggest that resveratrol maintained much of the shared microbial community while altering taxa that were either group-specific or differentially enriched between FD and treated mice.

## 4 Discussion

In this study, resveratrol alleviated FD-like pathology in mice, as shown by improved gastric emptying, reduced duodenal mucosal injury, and decreased mast cell accumulation. These changes were accompanied by alterations in immune-related transcriptional programs, epithelial function-related gene expression, and gut microbial structure. Resveratrol is a dietary polyphenol found in grapes, peanuts, berries, and other plant-derived foods (22). Previous studies have reported its antioxidant, anti-inflammatory, barrier-protective, and microbiota-modulating effects (26, 41, 42). These properties make resveratrol a potential candidate for the regulation of the altered gastroduodenal microenvironment in FD.

FD is a heterogeneous disorder involving impaired gastric motility, duodenal barrier dysfunction, low-grade inflammation, and changes in the gastroduodenal microbiota (3, 5, 16). In the present study, FD mice showed delayed gastric emptying together with villus injury, crypt disorganization, goblet cell alterations, submucosal edema, and inflammatory cell infiltration in the duodenal mucosa. Resveratrol treatment improved gastric emptying and alleviated these histological changes. These findings support the view that duodenal mucosal alterations may be closely associated with gastric dysfunction in FD rather than simply representing a secondary consequence of delayed gastric emptying (10, 16).

Mast cells are important effector cells in mucosal immune responses. Their mediators can influence epithelial permeability, neural sensitivity, and gastrointestinal motility (15, 43). Increased duodenal mast cell infiltration has also been reported in patients with FD and has been associated with impaired barrier integrity and symptom development (12). In the present study, mast cell numbers were increased in FD mice and decreased after resveratrol treatment. The changes in Kit, Mcpt1, and Cpa3 expression were consistent with the histological findings. Together, these results suggest that the effect of resveratrol was associated with reduced mast cell-related mucosal immune activation.

RNA-seq analysis showed that resveratrol treatment was associated with broad changes in immune-related transcriptional programs. The enriched functions were mainly related to mucosal immune responses, cytokine and chemokine signaling, innate immune sensing, and adaptive immune regulation. These results suggest that resveratrol did not affect only a single inflammatory pathway but was associated with broader regulation of the duodenal mucosal immune environment. The PPI network also connected immune-related genes, including Ccl2 and Il6, with genes associated with epithelial metabolism and extracellular matrix regulation, such as Slc34a2 and Has2.

The qPCR results further supported the RNA-seq findings. Resveratrol increased the expression of *Cyp24a1*, *Has2*, and *Slc34a2*, which are related to vitamin D metabolism, extracellular matrix remodeling, and epithelial transport, respectively (44–50). Resveratrol also altered the expression of genes associated with the candidate *Ccl2*–*Ccr2*, *Il6*–*Il6ra*/*Il6st*, and *Tnf*–*Tnfrsf1a* signaling axes. Additional changes in inflammatory, adaptive immune, antigen presentation, and epithelial barrier-related genes further indicated that treatment attenuated several transcriptional abnormalities observed in FD mice. However, these results represent changes in gene expression and do not directly demonstrate ligand–receptor binding or cell-specific communication. The regulation of *Ocln*, *Tjp1*, *Cldn1*, and *Muc2* suggests that epithelial barrier-related responses were also affected, although direct measurements of intestinal permeability were not performed.

Gut microbiota analysis showed clear differences in microbial community structure between the resveratrol-treated and FD groups. These differences were reflected in β-diversity, phylum-level composition, ecological assembly, diversity components, and predicted functional profiles. Previous studies have described a bidirectional relationship between resveratrol and the gut microbiota. Gut microorganisms participate in resveratrol metabolism, while resveratrol treatment may alter microbial composition and functional potential (27, 34, 51). In the present study, the predicted microbial functions were mainly related to nutrient metabolism, energy metabolism, and membrane transport. However, the resveratrol-treated group was also distinct from the control group, indicating that treatment did not simply restore the microbial community to a normal-like state. Therefore, these results should be interpreted as microbiota changes associated with resveratrol treatment rather than evidence that microbiota remodeling directly caused the improvement in FD-like pathology.

Several limitations should be acknowledged. First, this study did not include a healthy resveratrol-treated group. Therefore, it remains unclear whether resveratrol itself affects gastric emptying, immune-related gene expression, epithelial barrier-related genes, or gut microbiota composition under non-FD conditions. Future studies including healthy mice treated with resveratrol would help distinguish the disease-specific effects of resveratrol from its baseline physiological effects. Second, the findings were obtained from a mouse model and may not fully reflect the heterogeneity of human FD. Third, the immune- and barrier-related results were mainly based on transcriptional analysis. Protein-level validation, intestinal permeability measurements, and downstream functional experiments were not performed. Although resveratrol reduced mast cell accumulation and mast cell-related gene expression, histamine release and neural sensitization were not evaluated. In addition, the candidate chemokine- and cytokine-related signaling axes require further investigation using protein analysis, immunofluorescence co-localization, or cell-specific functional assays.

The 16S rRNA sequencing results were descriptive and did not directly measure microbial activity or establish a causal relationship between microbiota changes and improvement in FD-like phenotypes. Further studies using metabolomics, metagenomics, antibiotic depletion, or fecal microbiota transplantation may help clarify the biological role of the observed microbial changes. The optimal dose and duration of resveratrol treatment also remain to be determined.

In conclusion, resveratrol improved delayed gastric emptying, reduced duodenal mucosal injury, and decreased mast cell accumulation in FD mice. RNA-seq and qPCR analyses showed changes in immune-, epithelial-, and mucosal metabolism-related gene expression, while 16S rRNA sequencing identified marked differences in gut microbial structure after treatment. These findings suggest that the beneficial effects of resveratrol are associated with regulation of the duodenal mucosal microenvironment, although the underlying cellular mechanisms and the causal role of the gut microbiota require further investigation.

## Data Availability

The raw sequencing data generated in this study have been deposited in the National Center for Biotechnology Information (NCBI) Sequence Read Archive (SRA) under BioProject accession number PRJNA1480874. All other data supporting the findings of this study are available from the corresponding authors upon reasonable request. Requests for access to these data may be directed to Shanshan Cai (shanshancai1@outlook.com).

## Supplementary material

Supplementary Figure 1 Functional hierarchy of downregulated immune-related DEGs after resveratrol treatment. GO-DAG analysis was performed to visualize the hierarchical relationships among enriched GO terms for downregulated DEGs identified from RNA-seq analysis (n = 4 mice per group). **(A)** Biological process DAG analysis of downregulated DEGs; **(B)** cellular component DAG analysis of downregulated DEGs; **(C)** molecular function DAG analysis of downregulated DEGs.

Supplementary Figure 2 Functional hierarchy of upregulated mucosal defense-related DEGs after resveratrol treatment. GO-DAG analysis was performed to visualize the hierarchical relationships among enriched GO terms for upregulated DEGs identified from RNA-seq analysis (n = 4 mice per group). **(A)** Biological process DAG analysis of upregulated DEGs; **(B)** cellular component DAG analysis of upregulated DEGs; **(C)** molecular function DAG analysis of upregulated DEGs.
